# HMGB1, a potential regulator of tumor microenvironment in KSHV-infected endothelial cells

**DOI:** 10.3389/fmicb.2023.1202993

**Published:** 2023-07-13

**Authors:** Myung-Ju Lee, Joohee Park, Seokjoo Choi, Seung-Min Yoo, Changhoon Park, Hong Seok Kim, Myung-Shin Lee

**Affiliations:** ^1^Department of Microbiology and Immunology, Eulji University School of Medicine, Daejeon, Republic of Korea; ^2^Eulji Biomedical Science Research Institute, Eulji University School of Medicine, Daejeon, Republic of Korea; ^3^Department of Molecular Medicine, College of Medicine, Inha University, Incheon, Republic of Korea

**Keywords:** HMGB1, KSHV, herpesvirus, cell proliferation, CRISPR/Cas9 system, cytokine array

## Abstract

High-mobility group box 1 (HMGB1) is a protein that binds to DNA and participates in various cellular processes, including DNA repair, transcription, and inflammation. It is also associated with cancer progression and therapeutic resistance. Despite its known role in promoting tumor growth and immune evasion in the tumor microenvironment, the contribution of HMGB1 to the development of Kaposi’s sarcoma (KS) is not well understood. We investigated the effect of HMGB1 on KS pathogenesis using immortalized human endothelial cells infected with Kaposi’s sarcoma-associated human herpes virus (KSHV). Our results showed that a higher amount of HMGB1 was detected in the supernatant of KSHV-infected cells compared to that of mock-infected cells, indicating that KSHV infection induced the secretion of HMGB1 in human endothelial cells. By generating HMGB1 knockout clones from immortalized human endothelial cells using CRISPR/Cas9, we elucidated the role of HMGB1 in KSHV-infected endothelial cells. Our findings indicate that the absence of HMGB1 did not induce lytic replication in KSHV-infected cells, but the cell viability of KSHV-infected cells was decreased in both 2D and 3D cultures. Through the antibody array for cytokines and growth factors, CXCL5, PDGF-AA, G-CSF, Emmprin, IL-17A, and VEGF were found to be suppressed in HMGB1 KO KSHV-infected cells compared to the KSHV-infected wild-type control. Mechanistically, phosphorylation of p38 would be associated with transcriptional regulation of CXCL5, PDGF-A and VEGF. These observations suggest that HMGB1 may play a critical role in KS pathogenesis by regulating cytokine and growth factor secretion and emphasize its potential as a therapeutic target for KS by modulating the tumor microenvironment.

## 1. Introduction

High-mobility group box 1 (HMGB1) is a versatile protein that plays essential roles in normal cellular processes and pathological conditions, particularly inflammation and cancer (Martinotti et al., 2015; Wang and Zhang, 2020). As an immune protein released during tissue damage, infection, or inflammation, HMGB1 plays a crucial role in regulating innate and adaptive immune responses (Yang et al., 2020). In innate immunity, HMGB1 acts as a pro-inflammatory cytokine, stimulating the production of TNF-alpha and IL-1β, thereby promoting inflammation, immune cell recruitment, and activation for threat elimination (Lee S. A. et al., 2014). In adaptive immunity, HMGB1 enhances antigen presentation through dendritic cell receptor binding, facilitating antigen capture, processing, and presentation to T cells. This interaction drives T cell activation, proliferation, and differentiation into effector cells, shaping the adaptive immune response (Li et al., 2013). Inflammation is crucial for the development of tumors, and HMGB1 is a vital mediator of the inflammatory response. HMGB1 functions as an extracellular signaling molecule and stimulates the production of cytokines and chemokines, contributing to the pro-inflammatory response (Xu et al., 2016). Additionally, HMGB1 can bind to receptors on immune cells, promoting tumor growth and immune evasion (Zhang et al., 2019; Hubert et al., 2021). In cancer, HMGB1 is frequently overexpressed and is associated with poor patient prognosis (Zhang et al., 2015; Xu et al., 2016). HMGB1 influences multiple aspects of cancer progression, including DNA repair, transcription, angiogenesis, and metastasis (Tripathi et al., 2019; Wang and Zhang, 2020).

Kaposi’s sarcoma (KS) is a type of cancer that affects the endothelial cells lining blood vessels and is caused by the Kaposi’s sarcoma-associated herpesvirus (KSHV), also known as human herpesvirus 8 (HHV-8) (Boshoff et al., 1995; Ganem, 2010). KSHV promotes the development of KS by modulating various signaling pathways that regulate cell proliferation, survival, and angiogenesis (Watanabe et al., 2018). The virus encodes several proteins that activate or inhibit these pathways, leading to the uncontrolled growth of infected cells and the formation of KS lesions (Ganem, 2010). Furthermore, KSHV exploits multiple host genes to create a pro-inflammatory and pro-tumorigenic microenvironment that facilitates tumor growth and immune evasion (Lee M. S. et al., 2014; Jeon et al., 2019; Lee et al., 2023).

HMGB1 enhances KSHV replication and transcription activator (RTA) binding to RTA-responsive elements of KSHV target genes (Song et al., 2004). Furthermore, HMGB1 binds and synergistically upregulates the KSHV *ORF50* promoter in conjunction with RTA (Harrison and Whitehouse, 2008). In a previous study, we demonstrated that intracellular HMGB1 forms complexes with various proteins and that the levels of HMGB1-interacting proteins are altered during latent and lytic replication (Kang et al., 2021a). Furthermore, our findings indicated that extracellular HMGB1 enhances lytic replication, which correlates with viral production.

Our previous study demonstrated that HMGB1 has a crucial role in the replication of KSHV in KSHV-producing cancer cell line. However, the role of HMGB1 in the development of KS remains unclear. Although human endothelial cells are widely regarded as a source of KS spindle cells, the lack of primary human endothelial cells for gene depletion studies has hindered efforts to ascertain the specific functions of cellular proteins in KS pathogenesis. To address the limitations of the study exploring the involvement of HMGB1 in KS development using primary endothelial cells, we employed the CRISPR-Cas9 system to knock out (KO) HMGB1 in immortalized human endothelial cells known as HuARLT cells. Fortunately, we successfully isolated an HMGB1 KO clone from the HuARLT cells, as well as HMGB1 KO HuARLT cells that were infected with KSHV. Using these cell model, our findings revealed that HMGB1 was not essential for the proliferation of uninfected cells. However, in KSHV-infected cells, the absence of HMGB1 significantly inhibited cell proliferation in 2D culture and impaired sphere formation in 3D culture. Further analysis revealed differential expression of various cytokines and growth factors, including those involved in sphere formation and maintenance, in KSHV-infected cells lacking HMGB1. These results suggest that HMGB1 plays a critical role in KS development and that its absence can impair tumor growth and maintenance.

## 2. Materials and methods

### 2.1. Cell culture and reagents

iSLK BAC16 cells were cultured in Dulbecco’s Modified Eagle’s Medium (DMEM)/high glucose (Welgene, Gyeongsan, South Korea) containing 10% fetal bovine serum (FBS; GenDEPOT, Katy, TX, United States) and 1% antibiotic-antimycotic solution (Invitrogen, Waltham, MA, United States). HygromycinB (1.2 mg/mL; Invitrogen), geneticin (250 μg/mL; Invitrogen), and puromycin (1 μg/mL; Invitrogen) were added and the cells were cultured to maintain the latent infection of iSLK BAC16. HuARLT cells (May et al., 2010) were cultured in endothelial cell growth medium 2 (EGM-2, PromoCell, Heidelberg, Germany) supplemented with 2 μg/mL doxycycline (Sigma-Aldrich, St. Louis, MO, United States). All cells were cultured at 37°C in a humidified atmosphere containing 95% air and 5% CO_2_.

### 2.2. Virus isolation and infection

The iSLK BAC16 cells harboring recombinant KSHV BAC16 (Brulois et al., 2012) were used to produce virions. iSLK BAC16 cells were treated with 1.2 mM sodium butyrate (Sigma, Burlington, MA, United States) and 50 μg/mL doxycycline (Sigma) for 48 h to induce lytic replication. Upon the induction of lytic replication, DMSO was added to the culture media together with sodium butyrate and doxycycline at 0.1, 0.5%, or 1% of the total volume (Kang et al., 2021b). For virus isolation, the culture medium was collected and centrifuged at 300 × *g* for 10 min at 4°C to remove cell debris from the culture supernatant. The supernatant was centrifuged again at 2,000 × *g* for 10 min at 4°C, and the supernatant was collected. The supernatant was collected and centrifuged at 100,000 × *g* for 1 h at 4°C. The virus pellet was resuspended in cold phosphate-buffered saline (PBS) and stored at −80°C until the viral stock was used. KSHV infection was performed as previously described (Yoo et al., 2008). Briefly, the prepared KSHV stock was added to Gibco Opti-MEM (Invitrogen) containing 5 μg/mL polybrene (Santa Cruz Biotechnology, Santa Cruz, CA, United States). HuARLT cells were seeded onto 6-well culture plates the day before KSHV infection. KSHV infection was performed by centrifugation at 2,600 rpm for 1 h at 25°C. After centrifugation, the medium was changed to endothelial cell growth medium 2 (PromoCell), and the cells were incubated overnight at 37°C in a humidified atmosphere containing 5% CO_2_. The KSHV-infected cells were selected with 50 ~ 100 μg/mL of hygromycin B for over 2 weeks.

### 2.3. Establishment of HMGB1 KO cells using CRISPR/Cas9 system

HMGB-1 KO was performed according to a previously published method that targeted HMGB-1 (Kang et al., 2021a). To establish the HMGB-1 KO cell line, CRISPR RNA (crRNA) targeting HMGB-1 (catalog number A35509, chr13:30463559–30463537) and tracrRNA were purchased from Thermo Fisher Scientific and annealed according to the manufacturer’s instructions. Lipofectamine CRISPRMAX Cas9 transfection reagent (Thermo Fisher Scientific) was used to transfect HuARLT cells with CRISPR RNA and TrueCut Cas9 protein V2. After 3 days, the cells were detached with trypsin–EDTA and counted at the desired concentration. The cells were then seeded into a 96-well culture dish with 0.8 cells per well and 100 μL of medium for cloning. Single clones were analyzed by western blotting and sequencing to confirm HMGB-1 KO. The HMGB-1 KO clone was cultured in a large culture dish and used in subsequent experiments.

### 2.4. Sequencing to validate KO of HMGB1

To identify the KO cell clones and gene alterations using the CRISPR/Cas9 KO system, sequencing was performed as previously described (Kang et al., 2021a; Lee et al., 2023). DNA was extracted from HMGB-1 KO cells using the DNeasy Blood and Tissue kit (Qiagen, Valencia, CA, United States), and PCR was performed using a PCR premix (Solgent, Daejeon, South Korea) targeting HMGB-1. The following primers were used: F 5′-GAAAAATAACTAAACATGGGCAA-3′, and R 5′-GGAGGCCTCTTGGGTGCA-3′. The PCR products were validated by agarose gel electrophoresis and inserted into a vector using a TOPcloner TA kit (Enzynomics, Daejeon, South Korea). The resulting vector was transformed into *Escherichia coli* DH5α (included in the TA kit). Bacteria were cultured on LB plates containing 50 μg/mL ampicillin, and colonies were obtained. Ten colonies were selected, and plasmids were extracted using a Plasmid Mini kit (MGmed, Seoul, South Korea). The plasmids were sequenced by Bionics (Seoul, South Korea) using M13F (5′-GTAAAACGACGGCCAG-3′) and M13R (5′-CAGGAAACAGCTATGAC-3′) primers.

### 2.5. Western blotting

Western blot analysis was performed as previously described protocol (Jeon et al., 2021). The primary antibodies used were anti-HMGB-1 (Abcam, Cambridge, MA, United States), anti-β-actin (Sigma-Aldrich), anti-KSHV ORF 50 (Bioss, Woburn, MA, United States), anti-HHV ORF 45 (Thermo Fisher Scientific), and anti-KSHV K8.1 antibodies (Santa Cruz, Santa Cruz, CA, United States). Horseradish peroxidase (HRP)-conjugated goat anti-mouse IgG (Bethyl; Montgomery, TX, United States) and HRP-conjugated goat anti-rabbit IgG (Bethyl) were used as secondary antibodies. The antibody-reacted membranes were visualized using Amersham ImageQuant 800.

### 2.6. RNA isolation, cDNA synthesis, and reverse transcription-quantitative polymerase chain reaction (RT-qPCR) analysis

RNA was extracted using a TaKaRa MiniBEST Universal RNA Extraction Kit (Takara, Shiga, Japan) and adjusted to the same concentration per microliter. Reverse transcription was performed using TaKaRa RT Master Mix II (Takara) following the manufacturer’s instructions. The resulting complementary DNA was quantified using real-time PCR with TaKaRa SYBR FAST qPCR mix (Takara). qPCR was performed using the following primers: HMGB-1 F, 5′- GAA AAA TAA CTA AAC ATG GGC AA-3′ HMGB-1 R, 5′- CTA AGA AGT GCT CAG AGA-3′, glyceraldehyde 3-phosphate dehydrogenase (GAPDH) F, 5′-GGT ATC GTG GAA GGA CTC-3′, GAPDH R, 5′-GTA GAG GCA GGG ATG ATG-3′, ENA-78 F, 5′-CTG CAA GTG TTC GCC ATA GG-3′, ENA-78 R, 5′-GAG GCT ACC ACT TCC ACC TT-3′, PDGFA F, 5′-GTC ATT TAC GAG ATT CCT-3′, PDGFA R, 5′-TAA TTT TGG CTT CTT CCT-3′, G-CSF F, 5′-CAG AGC TTC CTG CTC AAG TG-3′, G-CSF R, 5′-TAG GTG GCA CAC TCA CTC AC-3′, Emmpirin F, 5′-AGG CTG TGA AGT CGT CAG AA-3′, Emmpirin R, 5′-GCC TCC TCA GAG TCA GT-3′, IL-17A F, 5′-TGT GAT CTG GGA GGC AAA GT-3′, IL-17A R, 5′-CCC ACG GAC ACC AGT ATC TT-3′, VEGF F, 5′-ATT ATG CGG ATC AAA CCT-3′, VEGF R, 5′-TTC TTG TCT TGC TCT ATC TT-3′, VEFGA F, 5′-AGG ATG GCT TGA AGA TGT-3′, VEGFA R, 5′-CAC GAAGTG GTG GTG AAG TTC-3′, VEGFC F, 5′-TGT GTC CAG TGT AGA TGA A-3′, VEGFC R, 5′-TCT TCT GTC CTT GAG TTG A-3′ KSHV ORF50 F, 5′-AGA AGG TGA CGG TAT ATC C-3′, KSHV ORF50 R, 5′-CGC TGT TGT CCA GTA TTC-3′, KSHV K8.1 F, 5′-AAC TGA CCG ATG CCT TAA-3′, KSHV K8.1 R, 5′-GCG TCT CTT CCT CTA GTC-3′. The primers used in this study were synthesized by Genotech (Daejeon, South Korea).

### 2.7. Immunofluorescence assay

Immunofluorescence assay (IFA) was performed as previously described (Lee M. S. et al., 2014; Kang et al., 2021a). The primary antibodies used were anti-HMGB-1 (Abcam), anti-KSHV ORF50 (Bioss), anti-KSHV K8.1 (Santa Cruz), and anti-LNA (Abcam) antibodies. The secondary antibodies used were Alexa Fluor 568-conjugated anti-mouse IgG (Thermo Fisher Scientific) and Alexa Fluor 568-conjugated anti-rabbit IgG (Thermo Fisher Scientific) antibodies. A concentration of 500 ng/mL of 49,6-diamidino-2-phenylindole (DAPI) was used to stain nucleic acids. The stained samples were observed under a Nikon Eclipse E400 microscope (Nikon, Tokyo, Japan) under the same conditions.

### 2.8. Proliferation and cell death assay

To assess cell proliferation, 5000 cells were seeded in each well of a 96-well plate and incubated for 1 day (under specified culture conditions). After incubation, the cells were treated with the WST-1 reagent (Roche, Basel, Switzerland) according to the manufacturer’s instructions. The plate was then incubated for 1 h (under specified culture conditions), after which absorbance was measured at 450 nm using a microplate reader. Three independent experiments were performed, and the data were statistically analyzed. To assess cellular death, 5000 cells were seeded in each well of a 96-well plate and incubated for 1 day (under specified culture conditions). The lactate dehydrogenase (LDH) assay kit (Roche) was used according to the manufacturer’s instructions. After the assay, absorbance was measured at 490 nm using a microplate reader. Three independent assays were performed, and the supernatant LDH levels were normalized to the cellular LDH levels. The data were statistically analyzed.

### 2.9. Three-dimensional culture of endothelial cell spheroid

Endothelial cells were cultured in a 3-dimensional culture system following a previously published method (Dubich et al., 2021; Lee et al., 2023). Briefly, 4000 endothelial cells were seeded in a 96-well plate coated with 1% agarose (Bio-Rad Laboratories, Hercules, CA, United States) in PBS. The cells were then cultured under adjusted growth conditions with doxycycline and hygromycin B for 2–3 days. The resulting spheroids were harvested from the wells and resuspended in 50 μL of endothelial cell growth media containing 2 μg/mL doxycycline, 0.7 mg/mL of human fibrinogen (Merck, Rahway, NJ, United States), 0.4% methylcellulose (Sigma-Aldrich), and 0.5 U/mL human plasma thrombin (Merck). The resuspended cells were mixed with 50 μL of Matrigel^®^ (BD Biosciences, Franklin Lakes, NJ, United States) and seeded in a 96-well plate. After polymerization, the plate was incubated under optimized cell culture conditions to allow further spheroid growth. To prevent drying, the plate was supplied with a culture medium containing doxycycline.

### 2.10. Cytokine antibody array

To assess cytokine production, 1 × 10^6^ endothelial cells were seeded onto 100 mm diameter dishes in an endothelial cell growth medium containing 2 μg/mL of doxycycline. After 2 days of culture, the supernatant was harvested and centrifuged at 2000 rpm at 4°C for 10 min to remove cellular debris. The supernatant was analyzed using the Proteome Profiler Human XL Cytokine Array Kit (R&D Systems, Minneapolis, MN, United States), following the manufacturer’s instructions. The final membrane was detected using Amersham^™^ ImageQuant^™^ 800.

### 2.11. Statistical analysis

All experiments were performed independently at least three times, and the results are representative data. The mean ± standard deviation values are presented in the graphs. A two-tailed Student’s *t*-test was used to compare the data between two different groups. Significant differences are indicated by an asterisk at a *p* value of less than 0.05 (*p < 0.05; **p < 0.01).

## 3. Results

### 3.1. Expression of HMGB1 in KSHV-infected human endothelial cells

**Figure 1:**
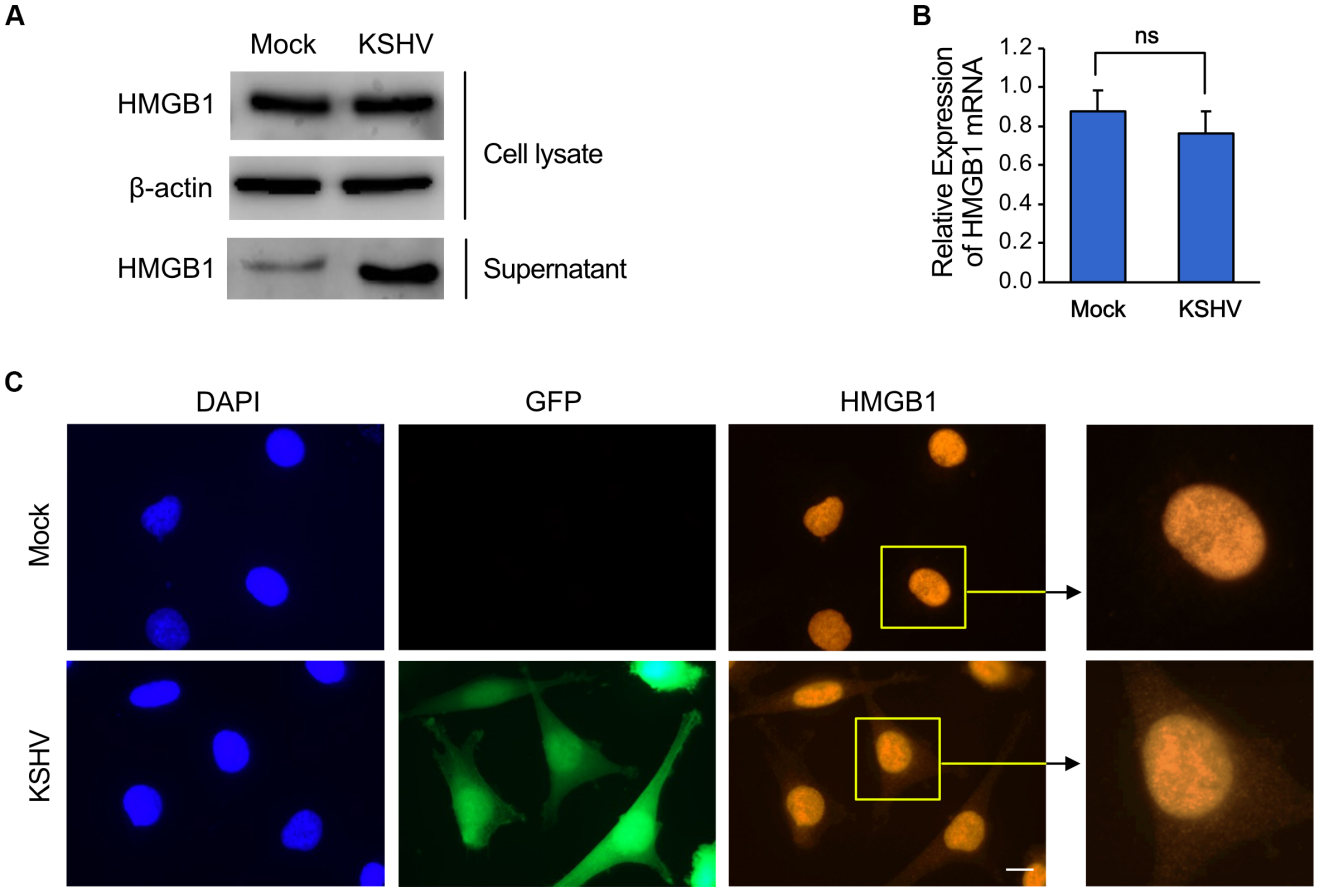
The expression of HMGB1 in KSHV-infected human endothelial cells. The immortalized human endothelial HuARLT cells were infected with KSHV, and the infected cells were isolated using hygromycin. The expression of HMGB1 in the KSHV-infected cells was compared with that of the mock-infected cells. **(A)** Western blot analysis for HMGB1 in the cell lysates and supernatant from the KSHV-and mock-infected cells. A representative blot is presented. β-actin was used as a house-keeping protein for normalization. **(B)** Densitometry analysis for the western blot results of **(A)**. Data are shown as the mean ± SD, *n* = 3, ns, not significant. **(C)** Immunofluorescence assay for HMGB1 in mock-and KSHV-infected HuARLT cells. KSHV BAC16 induces green fluorescence protein (GFP) in virus-infected cells. Scale bar, 10 μm.

We investigated whether KSHV infection affected the expression and secretion of HMGB1 in immortalized human endothelial HuARLT cells (Lipps et al., 2017). After infection with recombinant KSHV and KSHV BAC16 (Brulois et al., 2012), we selected KSHV-infected cells and found that HMGB1 expression in the cellular fraction was not significantly altered by KSHV infection (Figure 1A). However, we observed a significant increase in the protein level of HMGB1 in the supernatant of KSHV-infected cells compared with that in mock-infected cells (Figure 1A). This increase was not accompanied by an increase in HMGB1 mRNA expression (Figure 1B). Our results suggest that KSHV infection induces the translocation of HMGB1 from the nucleus into the cytoplasm of infected cells, and that this released HMGB1 may be secreted into the extracellular space. These findings were supported by immunofluorescence assays (IFA), which demonstrated HMGB1 expression in both the nucleus and cytoplasm of KSHV-infected cells but only in the nucleus of mock-infected cells (Figure 1C).

### 3.2. Establishment of an HMGB1 KO clone in human endothelial cells

**Figure 2:**
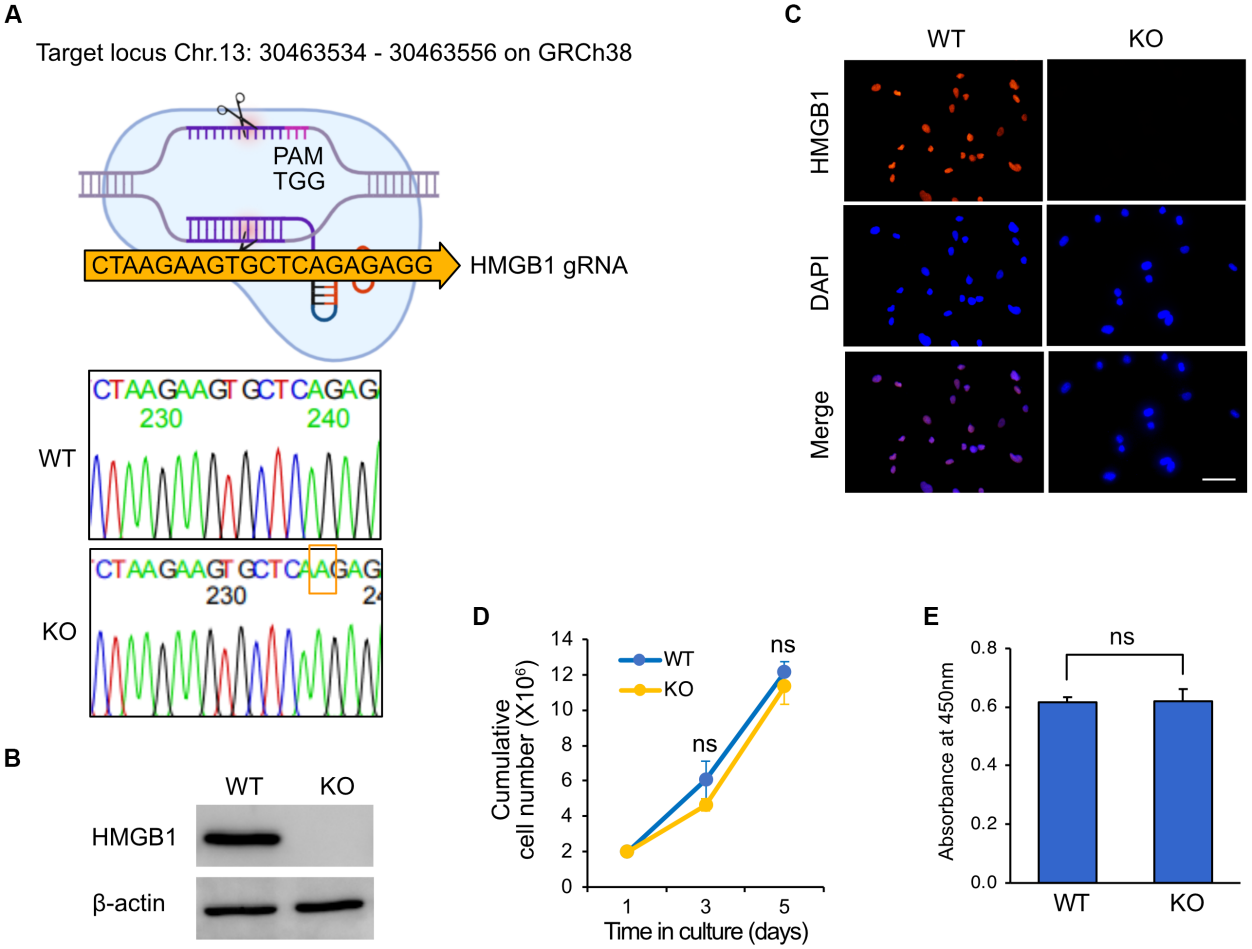
Knockout of HMGB1 in HuARLT cells. HuARLT cells were treated against HMGB1 using the CRISPR/Cas 9 system and gRNA against HMGB1. The isolated clone with a knockout (KO) of HMGB1 sequence, HMGB1 protein expression, and cell proliferation. **(A)** Schematic diagram showing CRISPR/Cas9 system with Cas9 targeting site of HMGB1. The PCR products containing gRNA targeting sequences from wild-type (WT) and KO clones were cloned into a T-vector, and the sequences were analyzed by conventional sequencing from 10 colonies. The nucleotide inserted into the CRISPR/Cas9 target site of HMGB1 in the KO clone is indicated by a red box. **(B)** Western blotting for HMGB1 expression in WT and HMGB1 KO cells. **(C)** Immunofluorescence assay for HMGB1 expression. Scale bar, 50 μm. **(D,E)** Proliferation of WT and HMGB1 KO iSLK BAC16 cells. At each time point, live cells were analyzed by trypan blue exclusion **(D)** and WST-1 viability testing **(E)**. Statistical significance of differences is indicated. ns, not significant, Student’s *t*-test.

To isolate a KO clone of *HMGB1* in HuARLT cells, we utilized a ribonucleoprotein complex composed of the Cas9 protein and *HMGB1*-targeting gRNA. To perform CRISPR/Cas9-mediated knockout, it is necessary to isolate a single cell clone by using limiting dilutions. However, this process can create cellular heterogeneity, which can make it difficult to interpret the results. To avoid this problem, a single clone was isolated from the HuARLT cells before the CRISPR/Cas9-mediated knockout process, and this clone was used for subsequent experiments. After transfection of gRNA and Cas9 protein to HuARLT cells, each clone was separated using a limiting dilution technique. To confirm HMGB1 KO, we employed a PCR-based genotyping method to detect genetic mutations. Subsequently, we cloned the PCR products, including the gRNA sequence, into a T-cloning vector to generate single-copy mutant DNA fragments for sequencing (Figure 2A). We analyzed 10 colonies using conventional sequencing analysis and found that all colonies exhibited the same mutation with an additional ‘A’ insertion in the Cas9 targeting site, indicating that both alleles might harbor the same mutation. We cannot exclude the possibility that only one allele was detected in the analysis using only 10 colonies. Intriguingly, the mutation induced by the gRNA sequence was the same as that in a previous application of iSLK BAC16 (ref), suggesting that the gRNA sequence might preferentially induce a mutation at a specific site. Furthermore, we analyzed HMGB1 expression in wild tyep (WT) and KO HuARLT clones using western blotting and IFA (Figures 2B,C). The cellular morphology and proliferation of KO clones were not significantly affected compared to those of WT cells, despite the HMGB1 ablation (Figures 2D,E).

### 3.3. KSHV gene expressions in HMGB1 KO human endothelial cells

**Figure 3:**
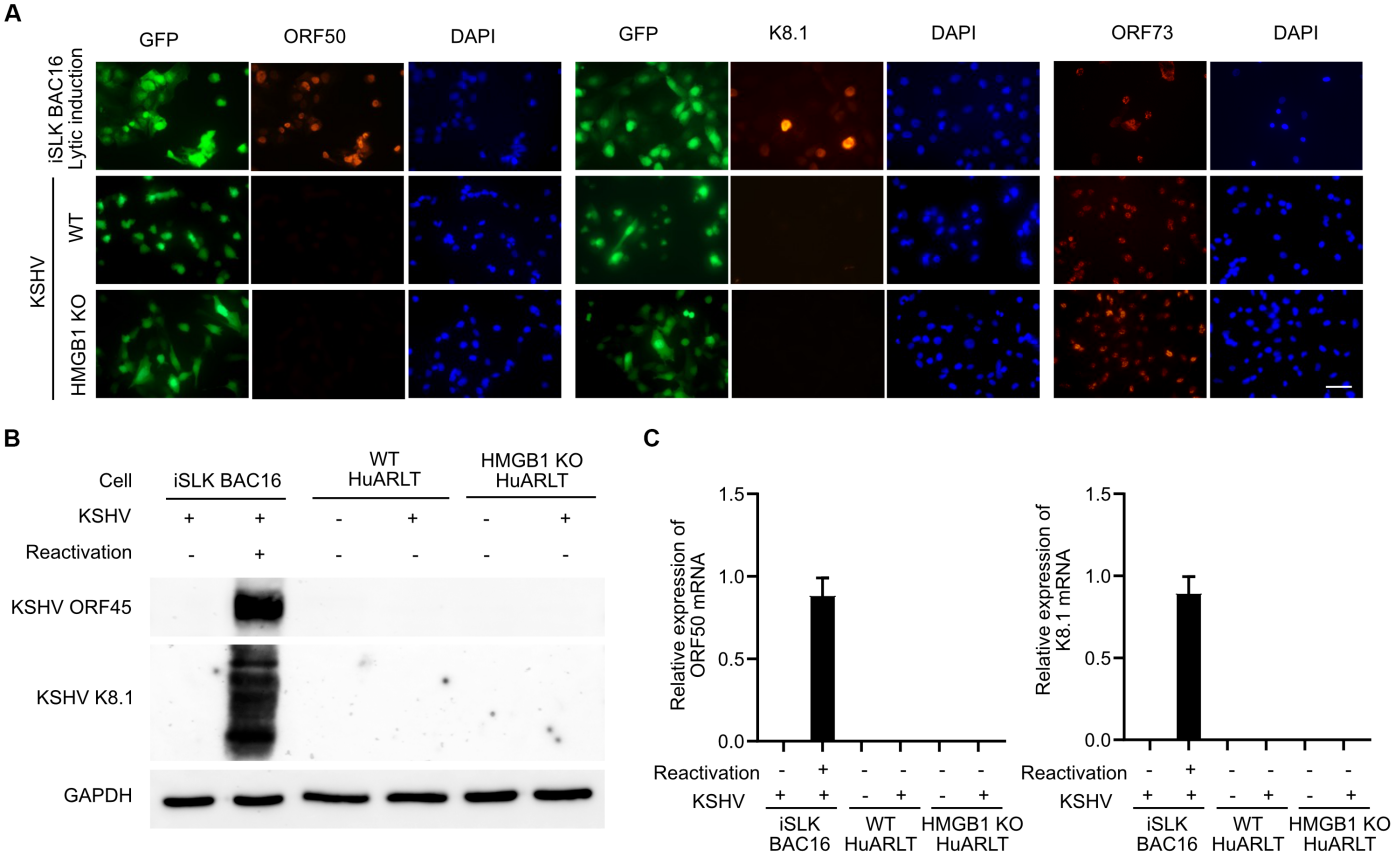
The expression of KSHV genes in WT and HMGB1 KO HuARLT cells. **(A)** Immunofluorescence assay (IFA) for KSHV ORF50, K8.1, and ORF73. iSLK BAC16 cells with reactivation were used as a positive control. Scale bar, 50 μm. **(B)** Western blot analysis for KSHV viral proteins. **(C)** Quantitative RT-PCR for KSHV mRNA expression. Data are shown as the mean ± SD, *n* = 3.

We infected an HMGB1 KO HuARLT clone with KSHV and investigated the expression of KSHV viral genes, including ORF50, K8.1, and ORF73 (Figure 3A). In the immunofluorescence assay (IFA), we found that while the latent gene ORF73 was detected in all KSHV-infected WT cells, the early lytic gene (ORF50) and late lytic gene (K8.1) were not detected, which is consistent with previous studies (Lee et al., 2023). In KSHV-infected HMGB1 KO cells, we did not observe any alteration in the viral gene expression of KSHV compared to that in KSHV-infected WT cells, indicating that both WT and HMGB1 KO cells showed latent infection in KSHV-infected cells. We obtained consistent results from western blotting and mRNA expression analyses by RT-qPCR, which supported the results of IFA (Figures 3B,C).

### 3.4. Cell viability of KSHV-infected WT and HMGB1 KO HuARLT cells

**Figure 4:**
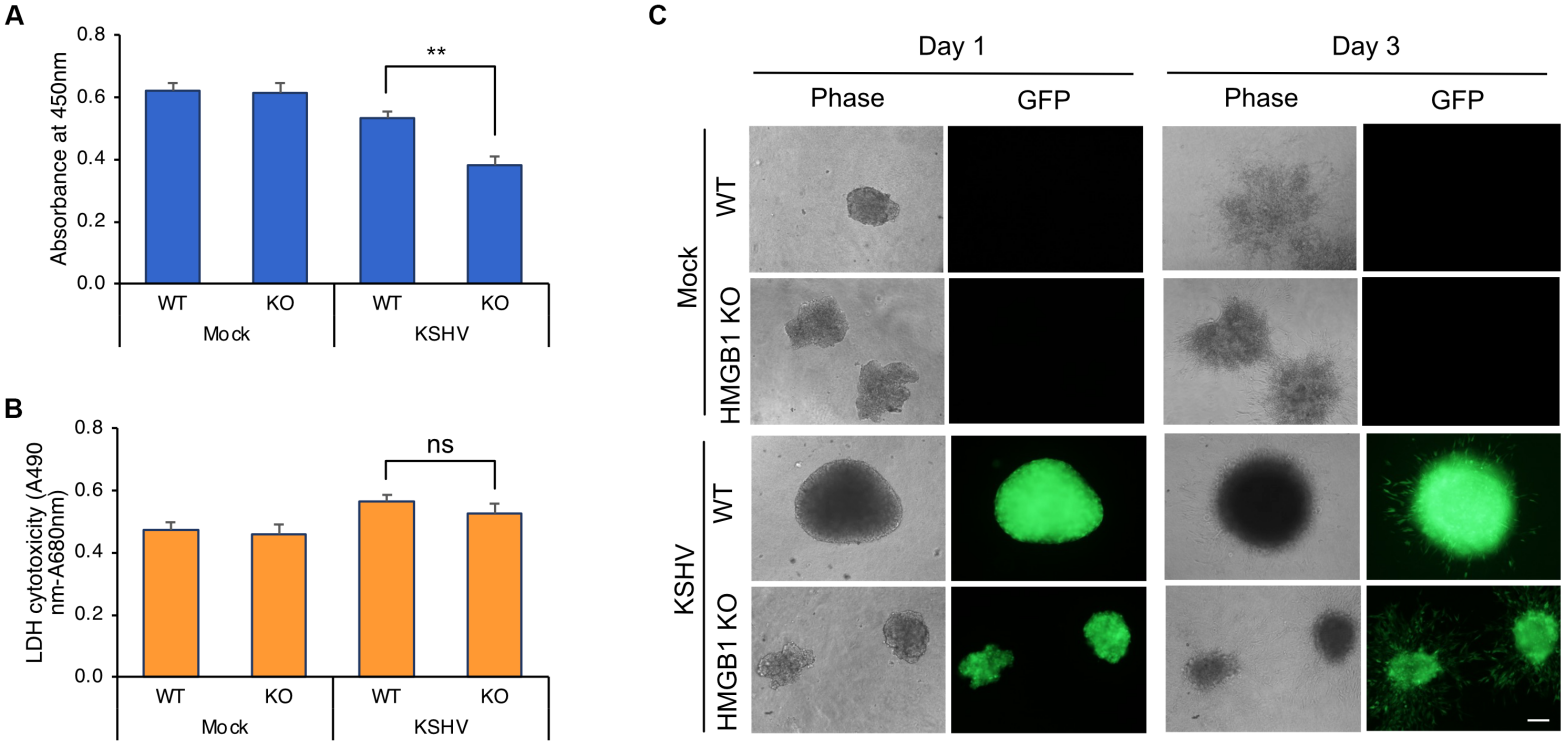
Cell viability and 3D culture of KSHV-infected HMGB1 KO HuARLT cells. **(A)** WST-1 cell viability assay. WT, wild-type HuARLT cells; KO, HMGB1 KO HuARLT cells; Mock, mock-infected cells; KSHV, KSHV-infected cells. **p < 0.01. **(B)** LDH assay. ns, not significant. **(C)** Sphere formation of cells in 3D culture. Day 1 and 3: the observation time after seeding the sphere on 3D culture. Scale bar, 100 μm.

The viability of KSHV-infected HMGB1 KO HuARLT cells was compared to that of KSHV-infected WT cells using the WST-1 cell viability assay (Figure 4A). While there was no significant difference in viability between mock-infected WT and HMGB1 KO cells, HMGB1 KO significantly affected the viability of KSHV-infected cells. HMGB1 KO resulted in lower viability of KSHV-infected cells than that of WT cells. The LDH assay was used to determine whether decreased cell viability was caused by increased cell death in HMGB1 KO cells compared to WT cells (Figure 4B). However, cell death was not significantly increased in HMGB1 KO cells following KSHV infection, indicating that the lower cell viability of KSHV-infected HMGB1 KO cells might be the result of a decrease in the cell proliferation rate. KSHV-infected HuARLT cells are known to induce sphere formation in 3D culture (ref). To investigate the effect of HMGB1 KO on sphere formation and maintenance, we induced the formation of spheres with mock-infected WT, mock-infected HMGB1 KO, KSHV-infected WT, and KSHV-infected HMGB1 KO cells (Figure 4C). KSHV-infected WT cells formed larger and more compact spheres than the mock-infected WT cells. Although HMGB1 KO did not significantly affect sphere formation in mock-infected cells, KSHV-infected HMGB1 KO cells showed smaller and less compact spheres than KSHV-infected WT cells. These results indicate that HMGB1 plays a vital role in sphere formation in 3D cultures and may be associated with tumorigenesis.

### 3.5. Cytokine, chemokine, and growth factor expression profile in WT and HMGB1 KO HuARLT cells infected with KSHV

**Figure 5:**
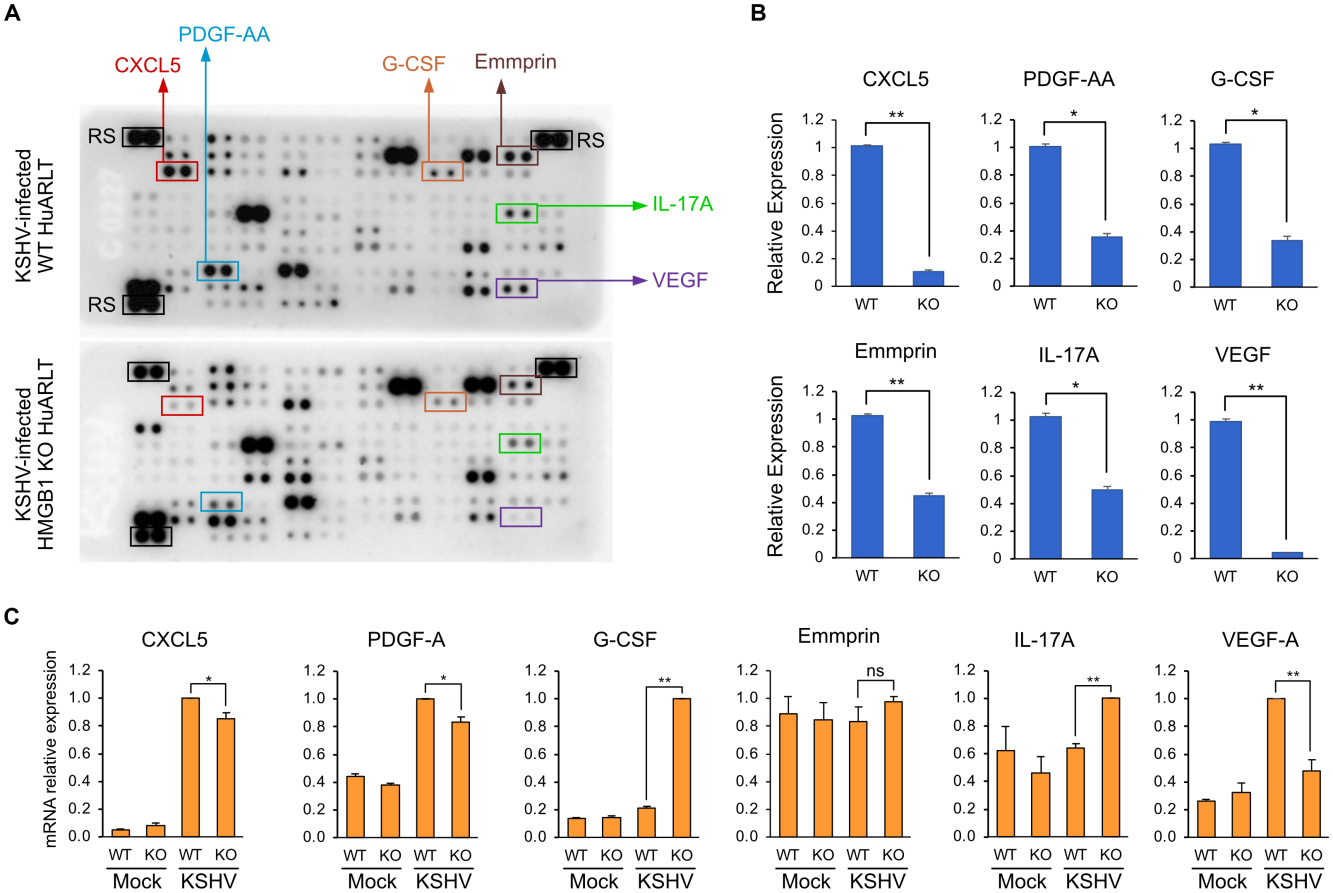
Cytokine expression levels in KSHV-infected HuARLT cells with HMGB1 KO. **(A)** Cytokine array. The conditioned media from KSHV-infected WT or HMGB1 KO cells were used for the cytokine array. The decreased proteins in HMGB1 KO cells were indicated as boxes. RS, reference spots as the experimental control. **(B)** Densitometry analysis for the selected proteins in cytokine array of **(A)**. Data are shown as the mean ± SD, *n* = 2, *p < 0.05, **p < 0.01. **(C)** Quantitative RT-PCR for the selected cytokines or growth factors. Data are shown as the mean ± SD, *n* = 3, **p < 0.01.

**Figure 6:**
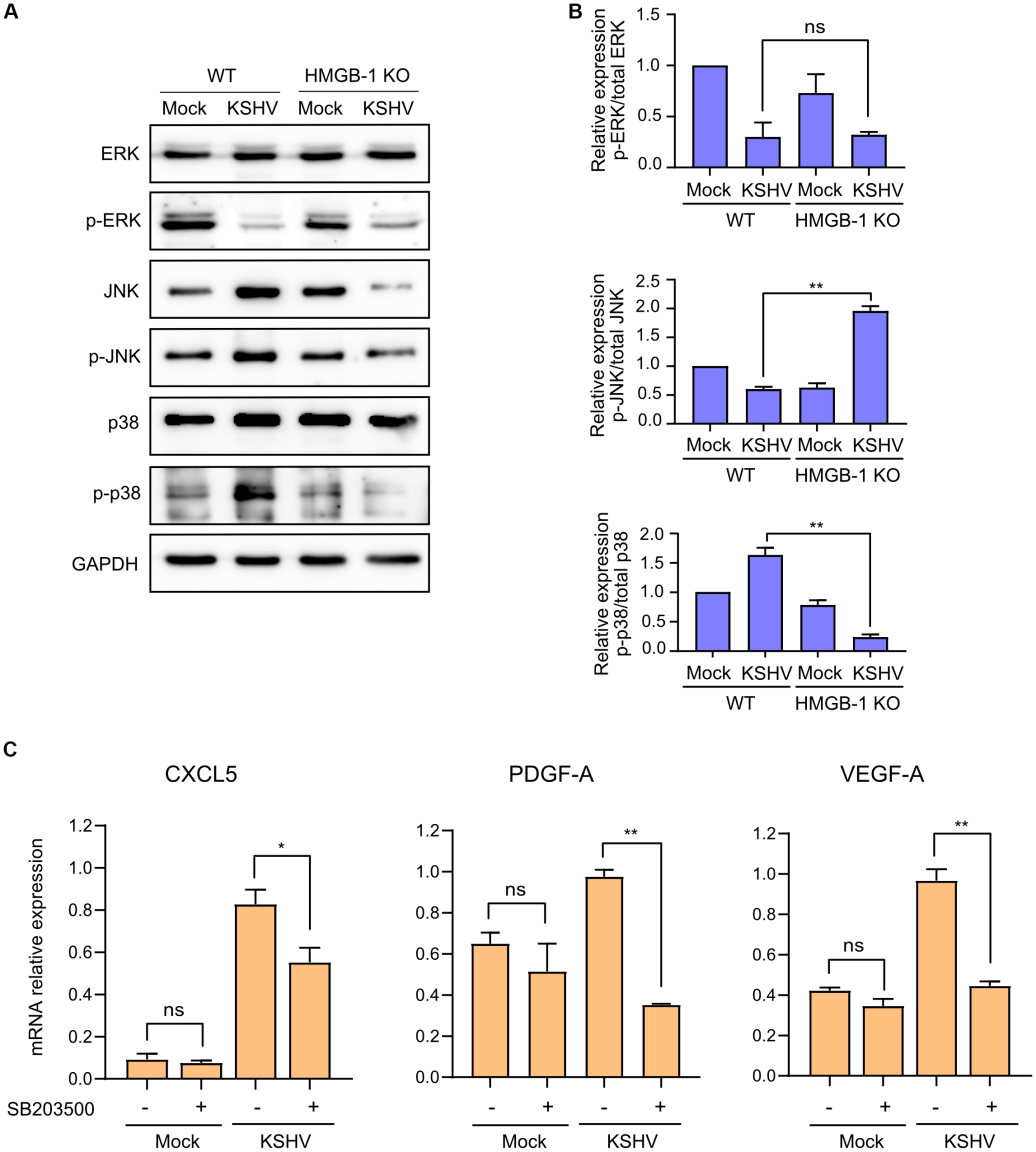
Signaling pathways related to KSHV-infected HMGB1 KO HuARLT cells. **(A)** Western blot analysis for HMGB1-associated signaling pathways. GAPDH was used as a housekeeping protein. **(B)** Densitometry analysis for western blotting results. Data are shown as the mean ± SD, *n* = 2, ns, not significant, **p < 0.01. **(C)** Relative expression of indicated mRNAs with chemical inhibitors for p38 pathways. SB203500 as a p38 inhibitor (2 μM) were treated in each cell for 24 h. DMSO treatment was used as a negative control. Each mRNA expression was analyzed by quantitative RT-PCR. Data are shown as the mean ± SD, *n* = 3, ns, not significant, *p < 0.05, **p < 0.01.

To investigate the changes in cytokine, chemokine, and growth factor expression in KSHV-infected HuARLT cells with HMGB1 KO, conditioned media were collected from each cell group and applied to a human cytokine antibody array. The array showed that some cytokines, chemokines, and growth factors were upregulated, whereas others were downregulated in HMGB1 KO cells (Figure 5A). This study focused on the downregulation of cytokines, chemokines, and growth factors by HMGB1 KO because cell proliferation was suppressed by HMGB1 KO (Figure 4). The cytokine array showed that CXCL5, PDGF-AA, G-CSF, Emmprin, IL-17A, and VEGF levels were significantly decreased in HMGB1 KO cells compared to those in WT cells (Figure 5B). For these poorly expressed factors in HMBG1 KO cells, mRNA expression analysis confirmed that CXCL5, PDGF-A, and VEGF levels were significantly decreased in HMGB1 KO cells compared to those in WT cells (Figure 5C). However, G-CSF, Emmprin, and IL-17A showed increased mRNA expression or no significant differences between KSHV-infected WT and HMGB1 KO cells. To examine the potential connection between certain proteins and a signaling pathway, HMGB1-related signaling pathways were analyzed by western blotting (Figures 6A,B). When HMGB1 was knocked out, we observed a significant reduction in p38 phosphorylation, suggesting a possible association between p38 and HMGB1 in KSHV-infected endothelial cells. In a previous study using iSLK BAC16 cells, we showed that the phosphorylation of JNK plays a critical role in KSHV replication regulated by HMGB1 (Kang et al., 2021a). However, in endothelial cells, although HMGB1 influenced the overall expression of JNK, we did not observe a suppression of the JNK phosphorylation by knocking out HMGB1. To investigate further, we employed chemical inhibitor targeting the p38 pathway (Figure 6C). By using SB203500, a p38 inhibitor, we examined the relationship between CXCL5, PDGF-A, VEGF-A, and this specific pathway in KSHV-infected cells (Figure 6C), and we found that it affected the mRNA expression of CXCL5, PDGF-A, and VEGF-A.

### 3.6. Glycyrrhizin inhibited the proliferation and maintenance of the 3D culture sphere in KSHV-infected endothelial cells mediated by HMGB1

**Figure 7:**
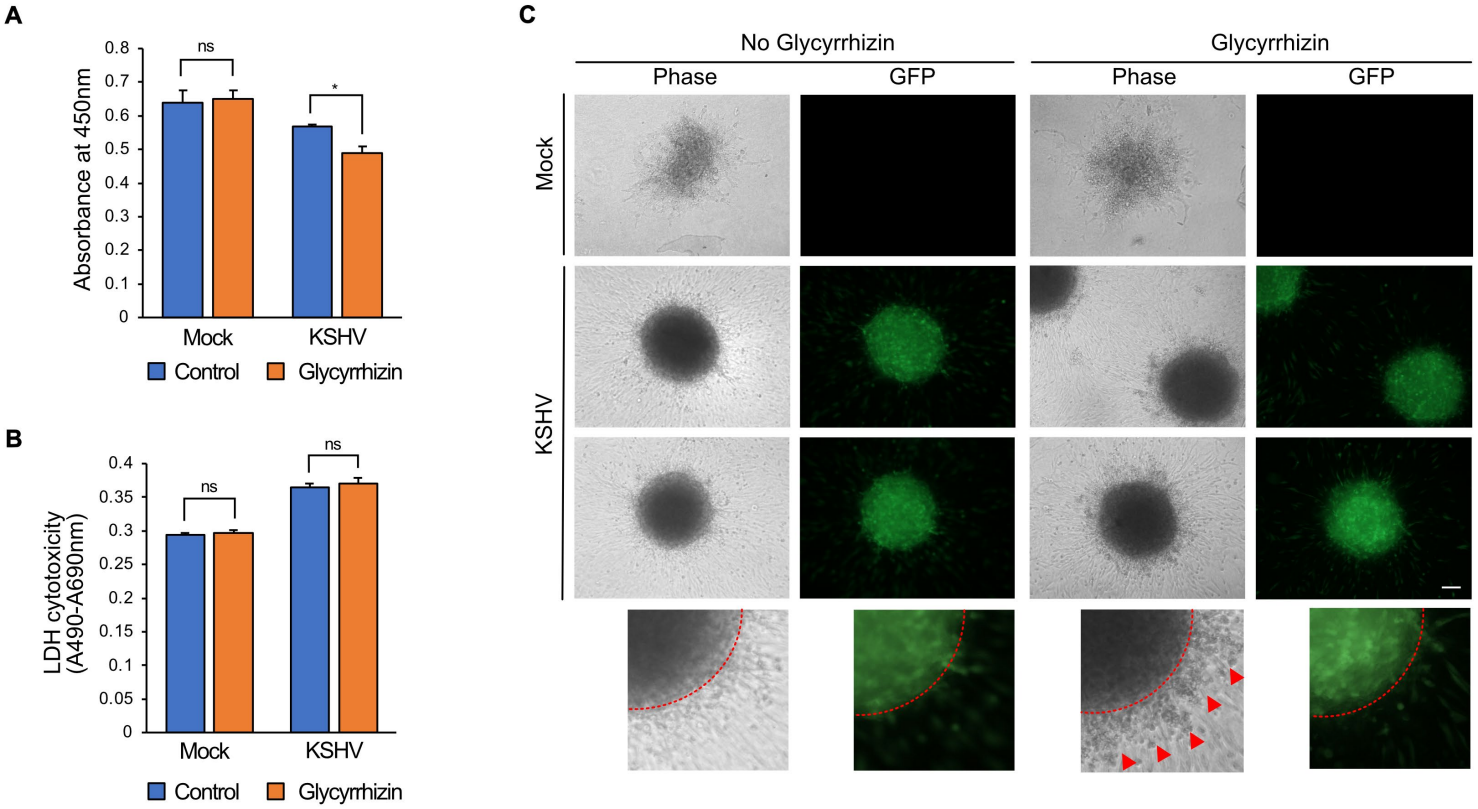
The effects of HMGB1 inhibitor, glycyrrhizin, on KSHV-infected HuARLT cells. **(A)** WST-1 cell viability assay for KSHV-infected HuARLT cells with or without glycyrrhizin. Data are shown as the mean ± SD, *n* = 3, ns, not significant, *p < 0.05. **(B)** LDH assay for KSHV-infected HuARLT cells with or without glycyrrhizin. Data are shown as the mean ± SD, *n* = 3, ns, not significant. **(C)** 3D culture sphere of mock-or KSHV-infected cells treated with glycyrrhizin. KSHV-infected cells expressed GFP. Red dotted line: the margin of the 3D culture sphere. Red arrows: dead cells or debris. Scale bar, 100 μm.

Glycyrrhizin is an extracellular HMGB1 inhibitor. When tested on mock-infected cells, no significant difference in cell viability was observed after the addition of glycyrrhizin. However, glycyrrhizin treatment significantly reduced the viability of the KSHV-infected HuARLT cells (Figure 7A). The LDH assay did not show any significant effects of glycyrrhizin on either mock-or KSHV-infected cells (Figure 7B), indicating that the decreased cell proliferation caused by glycyrrhizin was responsible for the suppressed viability of KSHV-infected cells. In 3D cultures, sphere formation was not significantly affected by glycyrrhizin in either mock-or KSHV-infected cells. However, treatment with glycyrrhizin led to an increase in dead cells and debris around KSHV-infected cells that did not express green fluorescence protein (GFP) (Figure 7C). These results suggested that HMGB1 may have different functions in 2D and 3D cultures.

## 4. Discussion

In our previous study, we established HMGB1 KO in the KSHV-producing cell line iSLK BAC16, in which HMGB1 KO decreased virion production by decreasing the expression of viral genes (Kang et al., 2021a). Several cellular viral proteins interacted with intracellular HMGB1 in the nucleosomal complex, and extracellular HMGB1 induced JNK phosphorylation to enhance the lytic replication of KSHV. Therefore, we demonstrated that HMGB1 plays a crucial role in the generation of infectious KSHV progeny during lytic replication. Since HMGB1 is closely associated with cancer development, we investigated the role of HMGB1 in the pathogenesis of Kaposi’s sarcoma (KS). While iSLK BAC16 is useful for producing recombinant KSHV, this cell line is not a suitable model for investigating KS development. Therefore, we established an HMGB1 KO cell line using immortalized human endothelial HuARLT cells.

HuARLT cells derived from HUVEC show tightly controlled proliferation with relevant phenotypic and molecular characteristics of endothelial cells via doxycycline-dependent regulation of two independent immortalizing genes (May et al., 2010). Because this cell line showed a KS-like phenotype with KSHV, it is a suitable model to investigate the pathogenesis of KSHV and a novel drug for the treatment of KS. Furthermore, we demonstrated that this cell line is useful for studying KSHV-host cell interactions by knocking out a target gene using the CRISPR/Cas9 system (Lee et al., 2023).

Many studies have been conducted on the role of HMGB1 in the regulation of cytokine, chemokine, and growth factor production. A previous study demonstrated that the inhibition of HMGB1 using glycyrrhizin led to a reduction in the expression of CCL2 and CXCL5, along with their respective receptors, CCR2 and CXCR2 (Wang et al., 2022). An association between HMGB1 and G-CSF has also been reported in previous studies (Yuan et al., 2020). HMGB1 administration restores blood flow recovery and capillary density by increasing VEGF expression in the ischemic muscles of diabetic mice (Biscetti et al., 2010). Consistently, we showed that HMGB1 KO suppressed CXCL5, G-CSF, and VEGF expression in the KSHV-infected cells. Additionally, we also found that PDGF-AA, Emmprin, IL-17A was suppressed by KO of HMGB1 in KSHV-infected endothelial cells. However, the protein expression and mRNA expression levels of these proteins did not display entirely consistent outcomes. The inconsistent results between protein and mRNA expression levels could be due to post-translational modifications or because mRNA expression was only analyzed at a specific time point in our experiments. Nevertheless, because the suppressed cytokines and growth factors in the cytokine array can promote cell proliferation, it is likely that multiple factors work in combination rather than as a single factor to mediate HMGB1-induced cell proliferation in KSHV-infected cells. Although the precise underlying mechanisms by which HMGB1 regulates these cytokines, chemokines, and growth factors are not fully understood, our findings provide insights into the role of HMGB1 in regulating cytokines, chemokines, and growth factors, suggesting that targeting HMGB1 could be a potential strategy for treating KS.

In this study, we found that six cytokines were downregulated in KSHV-infected cells following HMGB1 KO; however, their mRNA expression levels were not consistent with the results of the cytokine array. One possible reason for the lack of a correlation between mRNA expression and cytokine production in KSHV-infected cells is post-transcriptional regulation. Post-transcriptional regulation is a complex process involving various regulatory mechanisms, including microRNA-mediated regulation, which can modulate mRNA stability, translation, and protein synthesis, leading to discrepancies between mRNA expression and protein production. Another possible explanation is the involvement of other regulatory factors. HMGB1 is a multifunctional protein that interacts with various proteins and regulatory factors such as transcription factors, epigenetic modifiers, and signaling molecules. The downregulation of cytokine production in KSHV-infected cells after HMGB1 KO may be due to the disruption of the interaction between HMGB1 and other regulatory factors rather than the direct effect of HMGB1 on cytokine transcription. Although we showed that p38 pathway activated by HMGB1 was associated with VEGF expression, further studies are required to elucidate the exact underlying mechanisms.

This study has a specific limitation regarding its investigation of the role of HMGB1, as it was only conducted *in vitro*. HMGB1 is known to have diverse functions in both inflammation and the development of cancer. Therefore, it is important to recognize that the role of secreted HMGB1 may be more intricate within the microenvironment. To fully understand the precise impact of HMGB1 on KS development, further research using appropriate animal models specifically designed for KS would be necessary. Conducting such studies would provide more detailed insights into the exact contribution of HMGB1 to the development of KS and its implications in a living organism.

In summary, this study investigated the role of HMGB1 in KS, a cancer caused by KSHV infection. The results demonstrated that KSHV infection stimulated the secretion of HMGB1 in human endothelial cells, and that HMGB1 plays a critical role in KS pathogenesis by promoting cytokine and growth factor secretion and facilitating cellular sphere maintenance, suggesting that targeting HMGB1 could be a potential therapeutic strategy for treating KS.

## Data availability statement

The raw data supporting the conclusions of this article will be made available by the authors, without undue reservation.

## Author contributions

M-JL and HK designed the study. M-JL, JP, and SC performed the experiments. M-JL, S-MY, and CP analyzed the data. M-JL, JP, HK, and M-SL wrote the manuscript. All authors have read and approved the final version of the manuscript.

## Funding

This research was supported by a grant from the National Research Foundation of Korea (NRF), project number NRF-2022R1I1A3065538, to M-SL.

## References

[ref1] Biscetti F. Straface G. De Cristofaro R. Lancellotti S. Rizzo P. Arena V.. (2010). High-mobility group box-1 protein promotes angiogenesis after peripheral ischemia in diabetic mice through a VEGF-dependent mechanism. Diabetes 59, 1496–1505. doi: 10.2337/db09-1507 20200317 PMC2874711

[ref2] Boshoff C. Schulz T. F. Kennedy M. M. Graham A. K. Fisher C. Thomas A.. (1995). Kaposi's sarcoma-associated herpesvirus infects endothelial and spindle cells. Nat. Med. 1, 1274–1278. doi: 10.1038/nm1295-1274 7489408

[ref3] Brulois K. F. Chang H. Lee A. S.-Y. Ensser A. Wong L.-Y. Toth Z.. (2012). Construction and manipulation of a new Kaposi's sarcoma-associated herpesvirus bacterial artificial chromosome clone. J. Virol. 86, 9708–9720. doi: 10.1128/JVI.01019-12, PMID: 22740391 PMC3446615

[ref4] Dubich T. Dittrich A. Bousset K. Geffers R. Büsche G. Köster M.. (2021). 3D culture conditions support Kaposi's sarcoma herpesvirus (KSHV) maintenance and viral spread in endothelial cells. J. Mol. Med. (Berl) 99, 425–438. doi: 10.1007/s00109-020-02020-8 33484281 PMC7900040

[ref5] Ganem D. (2010). KSHV and the pathogenesis of Kaposi sarcoma: listening to human biology and medicine. J. Clin. Invest. 120, 939–949. doi: 10.1172/JCI40567, PMID: 20364091 PMC2847423

[ref6] Harrison S. M. Whitehouse A. (2008). Kaposi's sarcoma-associated herpesvirus (KSHV) Rta and cellular HMGB1 proteins synergistically transactivate the KSHV ORF50 promoter. FEBS Lett. 582, 3080–3084. doi: 10.1016/j.febslet.2008.07.055, PMID: 18692049 PMC7617400

[ref7] Hubert P. Roncarati P. Demoulin S. Pilard C. Ancion M. Reynders C.. (2021). Extracellular HMGB1 blockade inhibits tumor growth through profoundly remodeling immune microenvironment and enhances checkpoint inhibitor-based immunotherapy. J. Immunother. Cancer 9:e001966. doi: 10.1136/jitc-2020-001966, PMID: 33712445 PMC7959241

[ref8] Jeon H. Kang S. K. Lee M. J. Park C. Yoo S. M. Kang Y. H.. (2021). Rab27b regulates extracellular vesicle production in cells infected with Kaposi's sarcoma-associated herpesvirus to promote cell survival and persistent infection. J. Microbiol. 59, 522–529. doi: 10.1007/s12275-021-1108-6, PMID: 33877577

[ref9] Jeon H. Lee J. Lee S. Kang S. K. Park S. J. Yoo S. M.. (2019). Extracellular vesicles from KSHV-infected cells stimulate antiviral immune response through mitochondrial DNA. Front. Immunol. 10:876. doi: 10.3389/fimmu.2019.00876 31068945 PMC6491682

[ref10] Kang S. K. Kang Y. H. Yoo S. M. Park C. Kim H. S. Lee M. S. (2021a). HMGB1 knockout decreases Kaposi's sarcoma-associated herpesvirus virion production in iSLK BAC16 cells by attenuating viral gene expression. J. Virol. 95:e0079921. doi: 10.1128/JVI.00799-21, PMID: 34105998 PMC8312881

[ref11] Kang S. K. Lee M. J. Ryu H. H. Lee J. Lee M. S. (2021b). Dimethyl sulfoxide enhances Kaposi’s sarcoma-associated herpesvirus production during lytic replication. Front. Microbiol. 12:778525. doi: 10.3389/fmicb.2021.778525 34975802 PMC8716793

[ref12] Lee M. S. Jones T. Song D. Y. Jang J. H. Jung J. U. Gao S. J. (2014). Exploitation of the complement system by oncogenic Kaposi's sarcoma-associated herpesvirus for cell survival and persistent infection. PLoS Pathog. 10:e1004412. doi: 10.1371/journal.ppat.1004412, PMID: 25254972 PMC4177982

[ref13] Lee S. A. Kwak M. S. Kim S. Shin J. S. (2014). The role of high mobility group box 1 in innate immunity. Yonsei Med. J. 55, 1165–1176. doi: 10.3349/ymj.2014.55.5.1165 25048472 PMC4108799

[ref14] Lee M. J. Lee J. Kang S. K. Wirth D. Yoo S. M. Park C.. (2023). CXCL1 confers a survival advantage in Kaposi's sarcoma-associated herpesvirus-infected human endothelial cells through STAT3 phosphorylation. J. Med. Virol. 95:28020. doi: 10.1002/jmv.28020 35869037

[ref15] Li G. Liang X. Lotze M. T. (2013). HMGB1: the central cytokine for all lymphoid cells. Front. Immunol. 4:68. doi: 10.3389/fimmu.2013.00068, PMID: 23519706 PMC3602962

[ref16] Lipps C. Badar M. Butueva M. Dubich T. Singh V. V. Rau S.. (2017). Proliferation status defines functional properties of endothelial cells. Cell. Mol. Life Sci. 74, 1319–1333. doi: 10.1007/s00018-016-2417-5 27853834 PMC11107763

[ref17] Martinotti S. Patrone M. Ranzato E. (2015). Emerging roles for HMGB1 protein in immunity, inflammation, and cancer. Immunotargets Ther. 4, 101–109. doi: 10.2147/ITT.S58064 27471716 PMC4918250

[ref18] May T. Butueva M. Bantner S. Markusic D. Seppen J. MacLeod R. A.. (2010). Synthetic gene regulation circuits for control of cell expansion. Tissue Eng. Part A 16, 441–452. doi: 10.1089/ten.TEA.2009.0184, PMID: 19705962

[ref19] Song M. J. Hwang S. Wong W. Round J. Martinez-Guzman D. Turpaz Y.. (2004). The DNA architectural protein HMGB1 facilitates RTA-mediated viral gene expression in gamma-2 herpesviruses. J. Virol. 78, 12940–12950. doi: 10.1128/jvi.78.23.12940-12950.2004, PMID: 15542646 PMC524970

[ref20] Tripathi A. Shrinet K. Kumar A. (2019). HMGB1 protein as a novel target for cancer. Toxicol. Rep. 6, 253–261. doi: 10.1016/j.toxrep.2019.03.002 30911468 PMC6416660

[ref21] Wang P. Hao P. Chen X. Li L. Zhou Y. Zhang X.. (2022). Targeting HMGB1-NFkappab Axis and miR-21 by glycyrrhizin: role in amelioration of corneal injury in a mouse model of alkali burn. Front. Pharmacol. 13:841267. doi: 10.3389/fphar.2022.841267, PMID: 35586052 PMC9108160

[ref22] Wang S. Zhang Y. (2020). HMGB1 in inflammation and cancer. J. Hematol. Oncol. 13:116. doi: 10.1186/s13045-020-00950-x, PMID: 32831115 PMC7443612

[ref23] Watanabe T. Sugimoto A. Hosokawa K. Fujimuro M. (2018). Signal transduction pathways associated with KSHV-related tumors. Adv. Exp. Med. Biol. 1045, 321–355. doi: 10.1007/978-981-10-7230-7_15, PMID: 29896674

[ref24] Xu M. Zhou G. M. Wang L. H. Zhu L. Liu J. M. Wang X. D.. (2016). Inhibiting high-mobility group box 1 (HMGB1) attenuates inflammatory cytokine expression and neurological deficit in ischemic brain injury following cardiac arrest in rats. Inflammation 39, 1594–1602. doi: 10.1007/s10753-016-0395-2, PMID: 27363991

[ref25] Yang H. Wang H. Andersson U. (2020). Targeting inflammation driven by HMGB1. Front. Immunol. 11:484. doi: 10.3389/fimmu.2020.00484 32265930 PMC7099994

[ref26] Yoo S. M. Ahn A. K. Seo T. Hong H. B. Chung M. A. Jung S. D.. (2008). Centrifugal enhancement of Kaposi’s sarcoma-associated virus infection of human endothelial cells in vitro. J. Virol. Methods 154, 160–166. doi: 10.1016/j.jviromet.2008.07.026, PMID: 18755221

[ref27] Yuan S. Liu Z. Xu Z. Liu J. Zhang J. (2020). High mobility group box 1 (HMGB1): a pivotal regulator of hematopoietic malignancies. J. Hematol. Oncol. 13:91. doi: 10.1186/s13045-020-00920-3, PMID: 32660524 PMC7359022

[ref28] Zhang T. Guan X. W. Gribben J. G. Liu F. T. Jia L. (2019). Blockade of HMGB1 signaling pathway by ethyl pyruvate inhibits tumor growth in diffuse large B-cell lymphoma. Cell Death Dis. 10:330. doi: 10.1038/s41419-019-1563-8, PMID: 30988279 PMC6465275

[ref29] Zhang Z. Wang M. Zhou L. Feng X. Cheng J. Yu Y.. (2015). Increased HMGB1 and cleaved caspase-3 stimulate the proliferation of tumor cells and are correlated with the poor prognosis in colorectal cancer. J. Exp. Clin. Cancer Res. 34:51. doi: 10.1186/s13046-015-0166-1, PMID: 25986235 PMC4446854

